# Schwannoma of the external auditory canal: a case report

**DOI:** 10.1186/1746-160X-3-6

**Published:** 2007-01-15

**Authors:** Ozgul Topal, Selim S Erbek, Seyra Erbek

**Affiliations:** 1 Department of Otorhinolaryngology, Baskent University Faculty of Medicine, 06640 Bahcelievler, Ankara, Turkey

## Abstract

**Background:**

Schwannomas are uncommon benign tumors of the external auditory canal. The clinical features, the differential diagnosis, and the surgical treatment of these lesions are discussed.

**Case presentation:**

A 51-year-old patient presented with a mass obliterating the external auditory meatus. Excisional biopsy was performed. Diagnosis was reported to be schwannoma by histopathologic examination.

**Conclusion:**

Schwannoma, rarely seen in the external auditory canal, can be managed by a precise excision of the tumor via transmeatal approach.

## Background

Schwannomas are slow-growing benign tumors originating from the Schwann cells surrounding the peripheral, cranial or autonomic nerves. In head and neck region, schwannomas most commonly appear as acoustic neuroma (25–45%) [1]. These tumors are rarely diagnosed in the external auditory meatus [2,3].

## Case presentation

**Figure 1:**
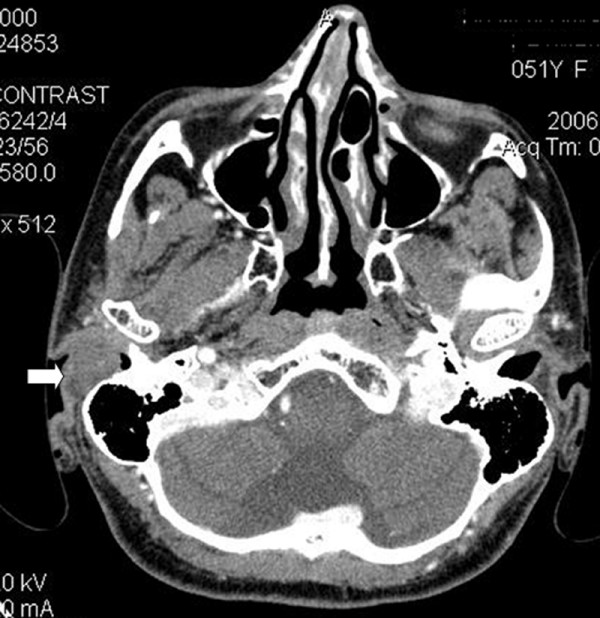
Contrast enhanced CT scan shows a soft tissue mass, 16 × 17 mm in size in the lateral part of the right external auditory canal posterior wall. Diagnosis was reported to be schwannoma by histologic examination.

A 51-year-old female patient presented with a mass originating from the posterior wall of the right external auditory meatus. She had a history of progressive right sided hearing loss and recurrent external otitis over a period of 12 months. Physical examination revealed an ovoid mass covered with normal skin without any pigmentation or ulceration. The mass completely obliterated the lateral half of the right external auditory meatus hindering the visualization of underlying tympanic membrane. Pure tone audiometry revealed a mild conductive hearing loss on the lesion side. Computer tomography (CT) scan of the temporal bone demonstrated a well-circumscribed soft tissue mass, 16 × 17 mm in size in the lateral part of the external auditory canal posterior wall (Fig. 1). The mass showed patchy contrast enhancement with no invasion of middle ear or surrounding bone/cartilaginous structures.

An excisional biopsy via transmeatal approach was performed under local anesthesia. The mass was totally removed while preserving the integrity of the overlying skin and the surrounding osteocartilaginous structures. At the end of the operation medial half of the canal wall and the tympanic membrane were seen to be intact and disease-free.

**Figure 2:**
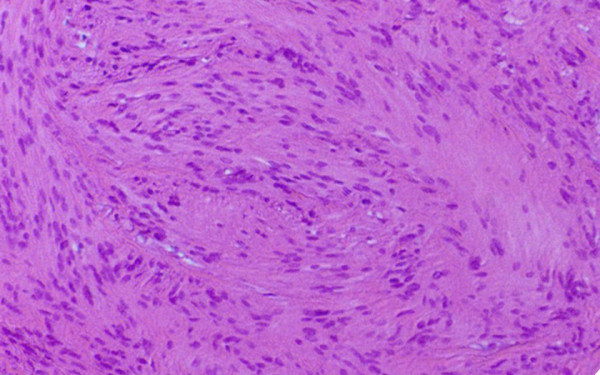
Histopathologic section of the tumor demonstrating areas of compact spindle cells arrayed in a palisade pattern called as Antony A. (H&E staining, original magnification ×10)

Diagnosis was reported to be schwannoma by histopathologic examination (Fig. 2). Schwann cells arranged in the 2 characteristic patterns referred to as Antoni A and B. Immunoperoxidase staining demonstrated a strong positivity to S-100 protein. There were no signs of local recurrence or narrowing of the external auditory canal during a 6 months of follow-up period.

## Discussion

Schwannomas are slow-growing benign tumors originating from the Schwann cells surrounding the peripheral, cranial or autonomic nerves. From a careful review of the literature, we found very few cases of schwannomas originating in the external auditory canal [2-6].

External auditory canal is innervated with the sensory branches of V, VII, IX, X and cervical plexus. In our case the mass was localized in the posterior and partly inferior canal wall, the sensory region of the greater auricular (C3) and lesser occipital nerves (C2, C3) respectively.

The clinical presentation of these tumors is usually with recurrent external otitis, pain and drainage with poor odour. Because of the enlarging mass, a mild conductive hearing loss is commonly encountered during the progression. Neurogenic symptoms such as pain or paresthesia are uncommon.

Schwannomas also can originate from the tympanic membrane [7]. In case of a mass obscuring the inspection of the inner parts of the external meatus, CT scan is very usefull in making decision about the extent of the lesion, integrity of the tympanic membrane and the type of the surgical approach.

Schwannomas consist of a true capsule facilitating the surgical dissection. They have a hard parenchymatous consistency with a non-infiltrating nature and exhibiting a smooth surface under a normal skin. Considering these morphological findings, the differential diagnosis should be made with respect to a number of other soft tissue neoplasms such as fibroma, chondroma, and leiomyoma. Definitive diagnosis should be based on the histological and immunohistochemical findings. Histologically, the tumor is characterized by streams of elongated spindle cells, with the elongated nuclei often arrayed in a palisade pattern. Areas consisting of thick concentration of cells are called Antoni type A (Verocay Body), whereas those in which the cells are loose and irregularly arranged are called as Antoni type B. A positive S-100 protein is the indicative of Schwann cell origin.

Neurofibroma also originates from Schwann cells and must be considered in histopathologic examination. Neurofibroma does not have a true capsule; have a dense cellular consistency and increased mitosis. Histologic sections show local invasion areas [8]. There is no Verocay Body formation. Neurofibromas are usually multicentric, which is an important clinical distinction from schwannomas. Neurofibromas may accompany with a special entity called as von Recklinghausen's disease (cafe-au-lait spots, neurofibromas, optic glioma, Lisch nodules, skeletal malformations, learning disabilities, bilaterally acoustic neuroma etc.). In this disease malign transformation is as high as 5–30% [9,10]. In our patient systemic evaluation was normal.

Treatment of choice in these tumors is excisional biopsy. Provided that the complete excision is performed, local recurrence is extremely rare. Transmeatal approach was performed in the present case while the good cleavage plane provided an en bloc resection with preservation of surrounding structures.

## Competing interests

The author(s) declare that they have no competing interests.

## Authors' contributions

O.T. drafted and prepared the manuscript. S.S.E. reviewed the patient's medical record in order to collect all the available information. S.E. was involved in revising the article for intellectual content details. All authors read and approved the final manuscript.
